# Attachment insecurity and psychological (mal)adjustment in older adults: The longitudinal role of fear of self‐compassion

**DOI:** 10.1111/papt.70059

**Published:** 2026-03-29

**Authors:** Lúcia Tavares, Paula Vagos, Ana Xavier

**Affiliations:** ^1^ Universidade Portucalense Infante D. Henrique (UPT), CINTESIS@RISE, CINTESIS.UPT Porto Portugal; ^2^ Departamento de Educação e Psicologia (DEP), William James Center for Research (WJCR) Universidade de Aveiro (UA) Aveiro Portugal; ^3^ Centro de Investigação em Neuropsicologia e Intervenção Cognitivo‐Comportamental (CINEICC), Faculdade de Psicologia e de Ciências da Educação da Universidade de Coimbra (FPCEUC) Coimbra Portugal

**Keywords:** attachment insecurity, fear of self‐compassion, mediation model, older adults, psychological (mal)adjustment, repeated‐measures design

## Abstract

**Objectives:**

Insecure attachment has been suggested to precede and perpetuate fear of self‐compassion, with a negative impact on mental health. However, this evidence was obtained using general‐age samples and cross‐sectional designs. Our objective was to, in an older adult sample, analyse longitudinally the indirect effect of fear of self‐compassion in the relationship between attachment insecurity and psychological (mal)adjustment, controlling for negative life events.

**Design:**

Our study consisted of a repeated‐measures design based on three data assessment moments across 6 months. Participants were 147 Portuguese community‐resident older adults.

**Methods:**

We conducted path analyses to test two models. Model A had attachment anxiety and attachment avoidance as independent variables, fear of self‐compassion as mediator and depressive symptoms and quality of life as dependent variables. Model B consisted of a respecified Model A with negative life events added as covariate.

**Results:**

Attachment anxiety and attachment avoidance predicted increased depressive symptoms fully through increased fear of self‐compassion. Attachment anxiety also predicted decreased quality of life partially through increased fear of self‐compassion. These mediation effects remained significant when negative life events were controlled for.

**Conclusions:**

In older adults, attachment insecurity underlies the fear of being self‐compassionate, which, in turn, leads to increased depressive symptoms and poorer quality of life over time. Fear of self‐compassion, therefore, should be taken into consideration when conducting research and/or intervention in this age‐group.

## INTRODUCTION

Scientific and technological progress has resulted in increased human life expectancy, and several modern societies show decreased birth rates and a progressively ageing population (Hantke et al., 2020). Portugal has one of the highest ageing rates in Europe and, as estimated by Statistics Portugal (INE) during the Census 2021, almost one quarter of the population (23.43%) was aged ≥65 years (INE, 2024).

Whereas it is possible to remain physically and mentally healthy, the ageing process normatively poses challenges such as diverse losses (e.g., spouse bereavement, cognitive decline, limitations in independence) and a greater vulnerability to illness and functional or sensorial incapacities. Adjusting to such negative life events can be challenging and may result in impoverished quality of life and mental health problems (Murayama et al., 2020). Indeed, older adulthood is associated with a relatively high prevalence of psychopathology, of which depression is one of the most frequent (Andreas et al., 2017). In older adults, depressive symptoms can be a response to losses and stress factors normatively associated with ageing, and the literature highlights this relationship between negative life events and depression (Kraaij et al., 2002; Zhang et al., 2017). Such events may also activate attachment behaviours (Van Assche et al., 2013).

According to the Attachment Theory (Bowlby, 1969, 1973, 1980), humans are born with an innate psychobiological and behavioural system, that is, the attachment system, that motivates them to seek proximity to others, that is, the attachment figures, who may protect and care for them when need. The attachment system starts being shaped during baby–caregiver(s) interactions and these social and affective experiences later result in individual differences in attachment security. Attachment figures who are consistently available and responsive foster a sense of secure attachment and the formation of positive working models, that is, mental representations of self and others. Securely attached individuals perceive themselves as worthy of love and care and perceive others as available and supportive. Attachment figures who are non‐supportive or inconsistent in their caregiving, however, make it difficult for a sense of security to be attained, often resulting in negative working models. Direct security seeking, that is, the primary attachment strategy, is often compromised in individuals with negative working models. This may, instead, foster the reliance on defensive secondary emotion regulation strategies (Bowlby, 1969, 1973, 1980; Shaver & Mikulincer, 2007).

Two major kinds of these strategies have been identified: hyperactivation and deactivation of the attachment system (Mikulincer & Shaver, 2003). Hyperactivation represents vehement efforts to engage with the attachment figure(s) and attempts to ensure their proximity, attention, and protection. Individuals using hyperactivation are often extremely sensitive to potential rejection or abandonment and tend to focus on personal inadequacies and threats to relationships. Deactivation is characterized by inhibited proximity‐seeking and by the suppression or disregard of circumstances that might activate the attachment system. Individuals resorting to deactivation strive to keep their distance from others and are often uncomfortable with closeness and intimacy, tend to avoid or suppress distressing thoughts and memories, and may be overreliant on personal strengths and independence (Mikulincer & Shaver, 2003, 2005; Shaver & Mikulincer, 2007). Accordingly, an individual's attachment orientation, that is, the systematic pattern of relational expectations, emotion and behaviours resulting from their history of interactions with attachment figures, can be defined along two dimensions: attachment anxiety and attachment avoidance. These represent, respectively, the extent to which an individual relies on hyperactivating strategies or deactivating strategies, and attachment security represents a low score in both dimensions (Brennan et al., 1998).

Research on attachment quality has consistently demonstrated that individuals with secure attachment orientations, compared with insecure orientations, generally exhibit more adaptive strategies of coping and emotion regulation, higher levels of beneficial psychological constructs, and lower levels of psychopathology (Cassidy & Shaver, 2008; Mikulincer & Shaver, 2016). However, although the attachment system remains active from birth to death (Bowlby, 1969, 1973, 1980), this evidence comes mostly from relatively young samples (Magai et al., 2016). In older adulthood, attachment insecurity has been associated with increased depression (Besser & Priel, 2008; Spence et al., 2018) and poorer quality of life (Bodner & Cohen‐Fridel, 2010; Platts et al., 2023). More research is needed to investigate specifically the mechanisms through which attachment insecurity impacts psychological (mal)adjustment in older adults. This has not, to our knowledge, been done before.

As a complement to the attachment system, Bowlby (1969, 1973, 1980) also proposed the existence of the caregiving behavioural system. Whereas the goal of the attachment system is to seek proximity and protection, the goal of the caregiving system is to provide for such needs. This conceptualization, in turn, can be broadened to encompass prosocial constructs such as compassion (Mikulincer & Shaver, 2005). As defined by the Dalai Lama (1995, pp. 61), compassion represents ‘an openness to the suffering of others with a commitment to relieve it’. Compassion can be further understood, according to Gilbert (2005, 2009), as a feeling or motivation for caregiving that recruits motives for care, emotion and cognitive competencies (e.g., attention and theory of mind) to be sensitive to others' needs. This innate motivation for compassion has three flows: for others, from others, and towards the self.

Research has shown that attachment security provides a foundation for (self)compassion and caregiving, as it may foster the appraisal of potentially distressing situations as manageable, mobilize positive emotions in difficult times, hold for a flexible adjustment to life circumstances, and provide a sense of personal agency (Gillath et al., 2005; Mikulincer & Shaver, 2005, 2016; Shaver et al., 2017). Nevertheless, there is also evidence that some individuals react negatively to positive emotions in general and to (self)compassion in particular. In implementing the Compassionate Mind Training program in a group of chronic mental health adult patients, Gilbert and Procter (2006) reported that some participants' first step towards self‐compassion was met with doubt, fear and resistance. These reactions stemmed from beliefs such as self‐compassion being a weakness, feeling unworthy of self‐compassion and yearning for love and kindness but also feeling lonely and rejected. This fear of self‐compassion can also be understood within the Attachment Theory. Namely, inadequate or inconsistent caregiving that gives rise to insecure attachment orientations may lead to fear responses to self‐compassion or beliefs of not deserving self‐compassion, leading to shifts towards positive and pleasant feelings being perceived as unfamiliar and frightening (Gilbert, 2005, 2010).

Accordingly, Gilbert et al. (2014) reported that, in a sample of depressed adult participants, fear of self‐compassion was positively correlated with insecure attachment. The authors suggested that insecurely attached individuals may have experienced, in the past, that seeking support from others was ineffective or unreliable, thus fostering fear towards gestures of care and self‐compassion. Likewise, Naismith et al. (2019), in a sample of adults with a personality disorder, reported that fear of self‐compassion was positively correlated with avoidant attachment. Furthermore, fear of self‐compassion was significantly predicted by a combination of adverse childhood events (e.g., parental invalidation). Finally, Matos et al. (2017), in a sample of adults from the general community, demonstrated that fear of self‐compassion mediated the relationship between early memories of safeness and warmth and depressive symptoms. The authors also discussed how the lack of affiliative memories may foster the fear of self‐compassion, which, in turn, impacts on depressive symptoms.

Taken together, these findings suggest that insecure attachment may precede and perpetuate fear of self‐compassion. Likewise, negative life events may exacerbate the individual's suffering (Murayama et al., 2020), which may then increase depressive symptoms and reduce quality of life. Notwithstanding, evidence regarding the impact of attachment quality in older adults is scarce (Magai et al., 2016). Additionally, studies investigating the relationship between attachment quality and fear of self‐compassion (Gilbert et al., 2014; Matos et al., 2017; Naismith et al., 2019) were cross‐sectional, limiting the scope of their findings. Finally, although some of these previous works recruited samples including older participants, to our knowledge, fear of self‐compassion has not yet been studied specifically in older adults.

The present study aimed to address this gap by analysing longitudinally the indirect effect of fear of self‐compassion in the relationship between attachment insecurity and psychological (mal)adjustment, controlling for negative life events. To this purpose, we used a sample of Portuguese community‐resident older adults and a repeated‐measures design with three data assessment moments, and conducted path analyses to test mediation models. Due to the scarce previous literature, our study was mostly exploratory. We hypothesized that attachment anxiety and attachment avoidance, assessed at time 1, would influence the fear of self‐compassion at time 2, which, in turn, would impact increased depressive symptoms and poorer quality of life at time 3. We further hypothesized that these mediation effects would remain significant when negative life events, assessed at all times, are controlled for.

## METHODS

### Study design

**FIGURE 1:**
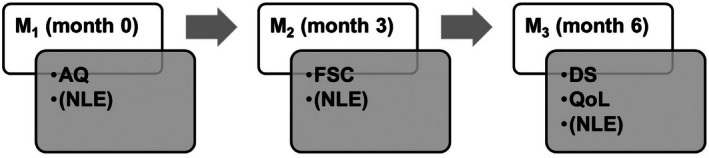
Data collection moments and assessed variables at each moment. M_1_ = assessment moment 1; M_2_ = assessment moment 2; M_3_ = assessment moment 3; AQ = attachment quality (i.e., attachment anxiety and attachment avoidance); FSC = fear of self‐compassion; DS = depressive symptoms; QoL = quality of life; NLE = negative life events.

We used a repeated‐measures design based on three data assessment moments across 6 months (Figure 1): moment 1 (M_1_; month 0), moment 2 (M_2_; month 3) and moment 3 (M_3_; month 6). The independent variables, attachment anxiety and attachment avoidance, were assessed at M_1_. The mediator variable, fear of self‐compassion, was assessed at M_2_. The dependent variables, depressive symptoms and quality of life, were assessed at M_3_. The covariate, negative life events, was assessed at all moments.

As outlined by Krull et al. (2016), repeated‐measures designs offer several methodological benefits that cross‐sectional studies lack. Specifically, collecting longitudinal data across multiple time points is essential for establishing the temporal precedence of variables, which, in turn, is crucial when testing mediation models.

### Procedure

The present study is part of a broader, ongoing research project and ethical approvals were obtained from the Ethics Committee for Health of Universidade Portucalense Infante D. Henrique, with the reference CES‐UPT‐01/05/21. The procedures used in this study adhere to the tenets of the Declaration of Helsinki. Participation was entirely voluntary and the participants did not receive any form of monetary or material incentive or compensation for their participation. All participants were given an informed consent form containing information regarding the project and their rights as participants.

Inclusion criteria were: age ≥65 years; resident in the community; no formal diagnosis of neurological and psychiatric disorders; and capacity to give informed consent. The sample was recruited from Universities for the Third Age and social centres in the north region of Portugal. At all three moments, data were collected using the pencil‐and‐paper versions of the instruments or an online version using the Google Forms platform, according to the participants' availability. Participants' responses were assigned an alphanumeric code to establish a correct association among all data collection moments, whilst maintaining response anonymity.

### Participants

G*Power 3.1 (Faul et al., 2009) was used to perform an a priori statistical power analysis for sample size estimation. With an alpha = .05 and power = .95, the projected sample size needed for an effect size = .25 is approximately *n* = 36 for within‐group comparisons and *n* = 132 for between‐factor comparisons. Based on this analysis, we decided to recruit an initial sample (M_1_) of at least 200 participants to account for dropout and to ensure adequate power effect.

Data from 263 participants was obtained at M_1_, from 185 participants at M_2_ for an attrition rate of 29.7% and from 147 participants at M_3_ for a further attrition rate of 20.5%. Such numbers are within what is reported in the literature, with attrition rates in longitudinal studies often ranging from 30% to 70% (Gustavson et al., 2012).

Only the data of participants who completed all three assessment moments was used in our analyses. This final sample consisted of 147 participants aged 65–92 years (*M* = 73.47, SD = 7.04). Years of formal education ranged from 2 to 25 years (*M* = 7.29, SD = 4.54). The majority of participants identified as female (*n* = 90, 61.2% females; *n* = 57, 38.8% males), were married (*n* = 94, 63.9% married; *n* = 35, 23.8% widowed; *n* = 12, 8.2% divorced; *n* = 5, 3.4% single; *n* = 1, 0.7% non‐marital partnership) and retired (*n* = 128, 87.1% retired; *n* = 16, 10.9% employed; *n* = 3, 2% unemployed).

Chi‐square tests of independence revealed that gender, *χ* ^2^(2) = 4.98, *p* = .083, civil status, *χ* ^2^(10) = 14.01, *p* = .172, and professional situation, *χ* ^2^(4) = 6.34, *p* = .175, did not significantly differ among participants who completed only the M_1_ assessment, participants who completed the M_1_ and M_2_ assessment, and participants who completed all three assessment moments. Likewise, eta correlation coefficients revealed that age (*η* = .02) and years of formal education (*η* = .04) had only a very weak association with the aforementioned assessment moments.

### Measures

#### Negative life events

The Negative Life Events Checklist (NLEC; Tavares et al., 2022) was created by the research team for the broader project in which the present study is included (for further information on this measure, please contact the first author). This checklist contains 14 items in a dichotomous *Yes/No* response format, which assess general events and events typical of old age and are summed to obtain a total score. Participants indicate if they experienced any of the presented events during the past month (e.g., ‘Being diagnosed with a new physical or mental condition’).

Psychometric work on this measure is underway but an exploratory factor analysis was already conducted using an independent older adult sample (Sousa, 2024), yielding the following fit indices: CFI = .96, TLI = .95, RMSEA = .028 (CIRMSEA = .000–.056, *p* = .892), SRMR = .162. Although the CFI and RMSEA values indicate that a one‐factor solution is relevant to understand negative life events in older adults, the SRMR value points to a meaningful part of that experience not being considered in this one‐factor solution. In that sample, good values were also found for composite reliability (.94) and test–retest reliability over 3 weeks (.46), as well as adequate convergent, divergent, and discriminant validity. In the present study, we conducted the Kuder–Richardson‐20 (KR‐20) test. At M_1_ and M_3_, the scale showed values of .62 and .60, respectively. At M_2_, however, the reliability value of .47 warrants some caution.

#### Attachment quality

The Adult Attachment Scale‐Revised (AAS‐R; Collins & Read, 1990) was used to assess attachment insecurity and contains 18 items rated on a 5‐point Likert‐format scale, varying from 1 (*Not at all characteristic of me*) to 5 (*Very characteristic of me*). Attachment anxiety and attachment avoidance were computed according to Collins (2008). The anxiety dimension (e.g., ‘I often worry that romantic partners don't really love me’.) assesses to what extent the individual worries about being abandoned or rejected, configuring a model of self. The avoidance dimension (e.g., ‘I find it difficult to allow myself to depend on others’.) assesses how comfortable the individual is with establishing close relationships and to what extent the individual believes they can rely on others, configuring a model of others.

The AAS‐R has shown adequate internal consistency (Cronbach's alpha, α = .69–.75), temporal stability (*r* = .52–.71) and convergent and discriminant validity (Collins & Read, 1990). The Portuguese version (Canavarro, 1997) has also shown adequate internal consistency (*α* = .69–.76) and test–retest reliability (*r* = .42–.65). In the present study, we found good internal consistency at M_1_: anxiety *α* = .93 and avoidance *α* = .89.

#### Fear of self‐compassion

The Fear of Compassion for Self subscale (FCSS), included in the Fears of Compassion Scales (FCS; Gilbert et al., 2011), was used to assess fear of self‐compassion. This subscale contains 15 items (e.g., ‘I fear that if I am more self‐compassionate I will become a weak person’.) rated on a 5‐point Likert‐format scale, varying from 0 (*Don't agree at all*) to 4 (*Completely agree*). Good values of Cronbach's alpha were reported in the original (.83; Gilbert et al., 2011) and Portuguese (.94; Matos et al., 2016) versions. In the present study, Cronbach's alpha assessed at M_2_ was also .94.

#### Depressive symptoms

The Geriatric Depression Scale‐15 items (GDS‐15; Sheikh & Yesavage, 1986) was used to assess depressive symptoms and contains 15 items in a dichotomous *Yes/No* response format (e.g., ‘Have you dropped many of your activities and interests?’). The GDS‐15 was created for specific use in older adults and was successful in differentiating depressed from non‐depressed individuals, proving to be a reliable screening tool while lessening response fatigue (Sheikh & Yesavage, 1986). In the Portuguese version of the GDS‐15 (Apóstolo et al., 2014), Cronbach's alpha = .83 and adequate construct validity have been reported. In the present study, the KR‐20 test conducted at M_3_ also demonstrated good reliability (.84).

#### Quality of life

The World Health Organization Quality of Life‐Older Adults Module (WHOQoL‐OLD; Power et al., 2005) was used to measure quality of life and contains 24 items (e.g., ‘Does the loss of sensory abilities affect your participation in activities?’) in a 5‐point Likert‐format scale rated differently according to certain subscales (e.g., ranging from 1, *Not at all*, to 5, *An extreme amount*). This original version allows for computing a total score and/or six facets (e.g., Sensory Abilities). Reported Cronbach's alpha for the total scale and the subscales range from .72 to .89 and satisfactory results regarding convergent, discriminant and construct validity have been shown (Power et al., 2005).

The Portuguese version of the WHOQoL‐OLD (Vilar et al., 2009) includes a culturally relevant Family/Family Life facet, for a total of 28 items. Good results were demonstrated regarding internal consistency (total scale α = .91), temporal stability (*r* = .80), and construct, convergent and discriminant validity. In the present study, a reliability analysis at M_3_ showed excellent internal consistency for the 28‐items total scale, α = .97.

### Data analysis

**FIGURE 2:**
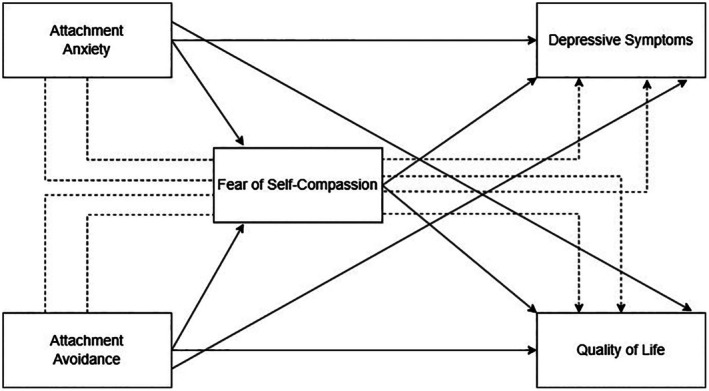
Path diagram for the conceptual model A. Solid lines represent direct paths; dash lines represent indirect paths.

IBM‐SPSS (v. 29; SPSS Inc., Chicago, IL) was used for preliminary data analyses and Mplus (v. 8.11; Muthén & Muthén) was used for the path analyses (for the respective syntax files, please see the Supplementary Material). Two models were tested and total, direct and indirect effects were analysed. Model A (Figure 2) had attachment anxiety and attachment avoidance measured at M_1_ as independent variables, fear of self‐compassion measured at M_2_ as mediating variable, and depressive symptoms and quality of life measured at M_3_ as dependent variables.

Subsequently, any non‐significant individual paths were progressively removed until a final model was achieved. Model B consisted of this final model with negative life events added as time‐varying covariates, thus accounting for fluctuations in exposure to negative life events across the six‐month period. Specifically, negative life events measured at M_1_ and at M_2_ were added as independent variables predicting the mediator and both dependent variables. Negative life events measured at M_3_ were added as an independent variable predicting both dependent variables.

To evaluate model fit, the comparative fit index (CFI), the Tucker–Lewis index (TLI), the root mean square error of approximation (RMSEA) with accompanying 90% confidence interval (CIRMSEA), and the standardized root mean square residual (SRMR) were used. Model fit criteria were chosen based on Hu and Bentler (1999): CFI and TLI ≥ .95, RMSEA ≤ .06 and SRMR ≤ .05 were considered a good fit.

Finally, to test mediation effects, a bootstrap procedure with 10,000 resamples was used to create 95% bias‐corrected confidence intervals around the standardized estimates of total, direct and indirect effects (Schumacker & Lomax, 2004). The effect is considered statistically significant (*p* < .05) if zero is not included on the interval between the lower and the upper bound of the 95% bias‐corrected confidence interval (Kline, 2005).

## RESULTS

First, we conducted a path analysis to test whether fear of self‐compassion mediated the relationship between attachment anxiety and attachment avoidance, depressive symptoms and quality of life (Model A).

**FIGURE 3:**
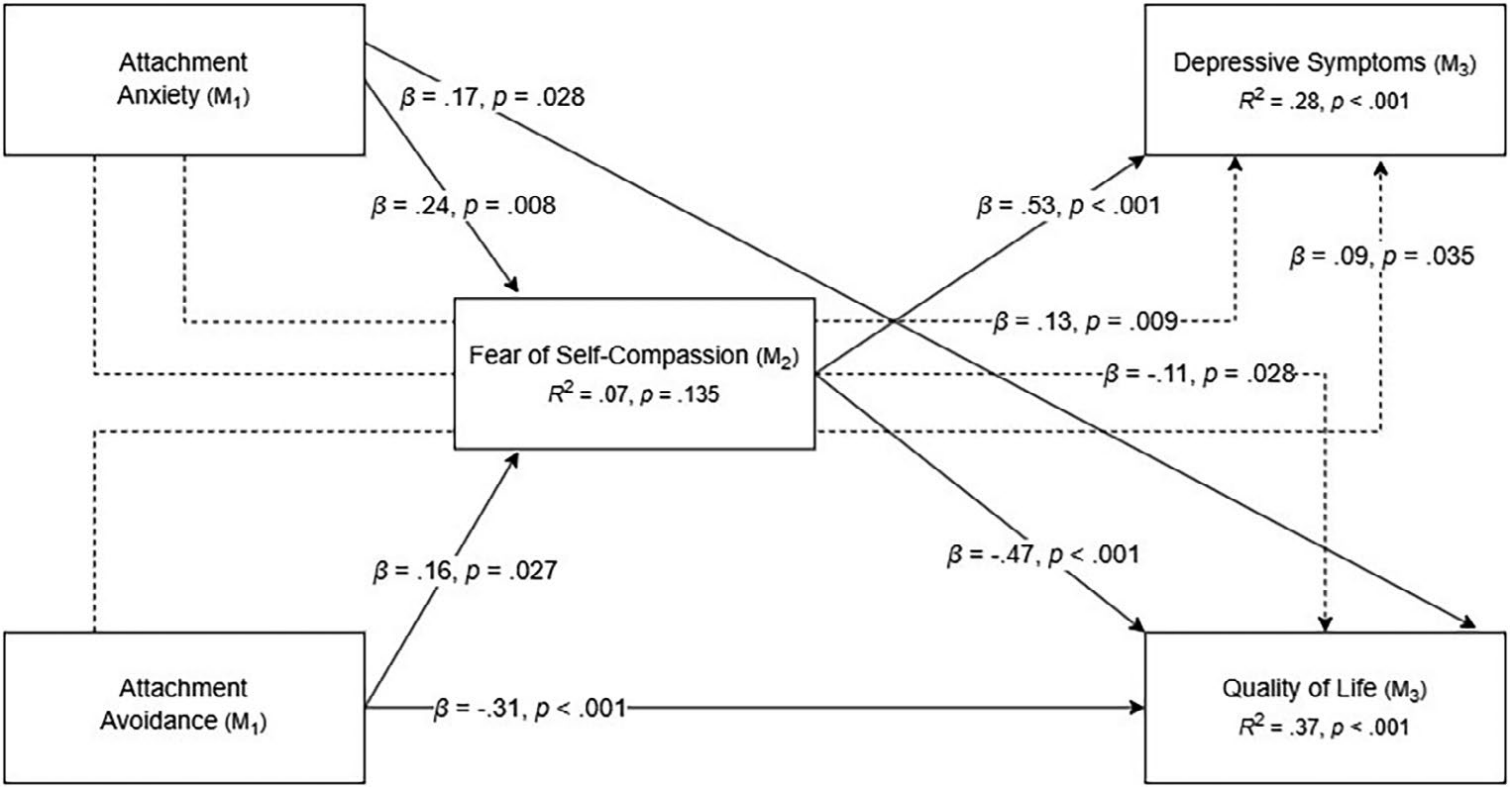
Path diagram for the final model A. Standardized path coefficients among variables are presented. Solid lines represent direct paths, dashed lines represent indirect paths. M_1_ = assessment moment 1; M_2_ = assessment moment 2; M_3_ = assessment moment 3.

In the initial saturated model, the direct path coefficient from attachment anxiety to depressive symptoms (*β* = .01, *p* = .951), first, and then the direct path coefficient from attachment avoidance to depressive symptoms (*β* = −.01, *p* = .876) were non‐significant. These paths were removed sequentially and the final model achieved a near‐perfect fit: CFI = 1.00, TLI = 1.00, RMSEA = .000 (CIRMSEA = .000–.000, *p* = .990), SRMR = .004. All the remaining direct paths were statistically significant. Figure 3 presents the path diagram for the final Model A and summarizes the information regarding direct paths, indirect paths and variance explained by this model.

Next, we analysed the indirect paths. Attachment anxiety and attachment avoidance predicted increased depressive symptoms through increased fear of self‐compassion. Because the direct paths from attachment anxiety and attachment avoidance to depressive symptoms were non‐significant, this is a full mediation effect.

Attachment anxiety also predicted decreased quality of life through increased fear of self‐compassion. Because the direct path from attachment anxiety to quality of life remained significant, this is a partial mediation effect.

Finally, the indirect path from attachment avoidance to quality of life via fear of self‐compassion was non‐significant (*β* = −.08, *p* = .058), that is, fear of self‐compassion did not mediate this relationship. The direct path from attachment avoidance to quality of life remained significant, that is, attachment avoidance impacted decreased quality of life directly.

The bootstrap resampling method confirmed the significance of all the aforementioned mediation effects, and the final model accounted for 28% and 37% of the variance of depressive symptoms and quality of life, respectively.

We then conducted a path analysis to test this final model while including negative life events (Model B). This model had a near‐perfect fit: CFI = 1.00, TLI = 1.00, RMSEA = .000 (CIRMSEA = .000–.059, *p* = .942), SRMR = .005. Two notes are of importance in analysing Model B. On the one hand, the following direct path coefficients were not statistically significant: from negative life events measured at M_1_ to fear of self‐compassion, depressive symptoms and quality of life; from negative life events measured at M_2_ to fear of self‐compassion, depressive symptoms and quality of life; and from negative life events measured at M_3_ to depressive symptoms and quality of life. On the other hand, all the direct and indirect paths previously found in the final Model A remained statistically significant, with one exception. In Model B, the direct path from attachment anxiety to quality of life became non‐significant (*β* = .15, *p* = .067). Therefore, in Model B, attachment anxiety predicted decreased quality of life fully through increased fear of self‐compassion (*β* = −.11, *p* = .046).

The bootstrap resampling method confirmed the significance of all the aforementioned mediation effects while controlling for negative life events, and this model accounted for 30% (*R* ^2^ = .30, *p* < .001) and 39% (*R* ^2^ = .39, *p* < .001) of the variance of depressive symptoms and quality of life, respectively.

## DISCUSSION

The impact of attachment quality on mental health is well demonstrated (Cassidy & Shaver, 2008; Mikulincer & Shaver, 2016), although such findings were mostly obtained in relatively young samples (Magai et al., 2016). Nonetheless, the attachment system remains relevant in older adulthood given the increased likelihood of experiencing negative life events, which may, in turn, negatively impact psychological adjustment (Van Assche et al., 2013). More research with robust designs is needed to further investigate attachment quality and related constructs in older adults. Additionally, fear of self‐compassion has been associated with early experiences in threatening environments and inadequate caregiving, leading to positive feelings being perceived as unfamiliar and frightening (Gilbert, 2005, 2010). The literature suggests that insecure attachment may precede and perpetuate fear of self‐compassion, although such findings come from general‐age samples and cross‐sectional designs (Gilbert et al., 2014; Matos et al., 2017; Naismith et al., 2019).

The present study intended to address these issues and it extends this literature by being, to our knowledge, the first to examine these relationships longitudinally and specifically among older adults. Using a sample of Portuguese community‐resident older adults and a repeated‐measures design with three data assessment moments, we investigated longitudinally the indirect effect of fear of self‐compassion in the relationship between attachment insecurity and psychological (mal)adjustment, controlling for negative life events. This longitudinal approach provides a more rigorous test of temporal and directional effects than cross‐sectional designs, offering stronger evidence for the proposed mediational pathways. In general, results supported the mediating role of fear of self‐compassion and confirmed our exploratory hypotheses.

Regarding direct effects, attachment anxiety and attachment avoidance predicted increased fear of self‐compassion 3 months later. This is in line with, and expands on, previous cross‐sectional findings (Gilbert et al., 2014; Matos et al., 2017; Naismith et al., 2019). It seems that, in Portuguese older adults, the tendency to worry about being abandoned by significant others, that is, attachment anxiety, and the difficulty to depend on and be close to others, that is, attachment avoidance, both lead to higher fear of self‐compassion. Said results can be contextualized within the Attachment Theory (Bowlby, 1969, 1973, 1980) and the Social Mentalities Theory (Gilbert, 2000). Individuals high in attachment anxiety may struggle to be self‐compassionate because they view themselves as undeserving of love and care (Gillath et al., 2005). Individuals high in attachment avoidance struggle to see others as trustworthy figures and may have been punished, shamed, or invalidated in the past when seeking care and help (Liotti, 2004), leading to (self)compassionate behaviours being perceived as threatening and aversive (Gillath et al., 2005).

Furthermore, fear of self‐compassion directly predicted increased depressive symptoms and lower quality of life 3 months later. This is also in line with previous cross‐sectional studies in general‐age samples associating fear of self‐compassion with detrimental mental health outcomes (Gilbert et al., 2014; Matos et al., 2017; Naismith et al., 2019). To our knowledge, our study is the first to demonstrate such impact of fear of self‐compassion on the psychological (mal)adjustment of older adults.

Regarding indirect effects, attachment anxiety and attachment avoidance predicted increased depressive symptoms fully through increased fear of self‐compassion. This confirmed our first hypothesis and, again, expanded on previous findings (e.g., Matos et al., 2017). Contextualizing said results also within the Attachment Theory and the Social Mentalities Theory, the lack of positive affiliative experiences as a child may lead to the development of insecure attachment, and negatively impact emotion regulation and the ability to generate warmth and feel safe within social relationships (Gilbert, 2005, 2010). This may result in conditioned memories where the need for soothing and care becomes associated with negative emotions and punishment or neglect by the attachment figure(s), therefore fostering the perception of care for the self as aversive. Such a mindset would lead the individual to avoid engaging in self‐compassion which, in turn, may exacerbate depressive feelings (Gilbert, 2005, 2009; Matos et al., 2017). Our results, therefore, clarify how attachment insecurity in Portuguese older adults may impact heightened depressive symptoms over time.

Regarding the impact on quality of life, attachment anxiety predicted decreased quality of life partially through increased fear of self‐compassion (Model A). However, when negative life events were added to the analysis (Model B), the mediation effect remained significant but the direct path from attachment anxiety to quality of life became non‐significant. An explanation may be that quality of life was generally assessed based on contextual factors (e.g., impact of sensorial deficits, opportunities for autonomous decisions and plans, participation in community activities; Vilar et al., 2009). It seems plausible that the perception of quality of life can be influenced by the subjective experience of negative life events. For example, being diagnosed with a health condition or incapacity may negatively impact the individual's autonomy and opportunity to engage in meaningful activities, leading to impoverished quality of life. Hence, adding these individual experiences to the model may have been sufficient to lessen the direct effect of attachment anxiety on quality of life. Notwithstanding, these results also confirmed our first hypothesis. In Portuguese older adults, attachment anxiety, that is, a negative model of self, may lead to increased fear of developing a self‐compassionate mindset, which, in turn, results in impoverished quality of life over time.

Additionally, there was a negative and statistically significant direct path from attachment avoidance to quality of life, indicating that attachment avoidance predicted decreased quality of life 6 months later. This, again, corroborates and expands on previous literature (Bodner & Cohen‐Fridel, 2010; Platts et al., 2023). Our findings suggest that, in Portuguese older adults, feeling uncomfortable with establishing close and intimate relationships and feeling unsure of whether others can be relied on predicts impoverished quality of life over time.

Finally, our second hypothesis was also confirmed, in that the three aforementioned mediation effects remained significant when negative life events were controlled for. The relationship between negative life events and poor mental health in older adults is well established (Kraaij et al., 2002; Zhang et al., 2017), and the present study demonstrated that, even when controlling for these effects, attachment insecurity negatively impacted psychological adjustment over time through increased fear of self‐compassion.

### Limitations and future research

The present study is not without limitations. Despite the robust research design with three data assessment moments, the total duration of the study was relatively short (i.e., 6 months). We only analysed the role of fear of self‐compassion and did not consider the other two flows, that is, compassion towards others and compassion received from others (Gilbert, 2005, 2009, 2010). Our eligibility criteria were broad, allowing us to recruit a diverse sample regarding characteristics such as formal education, but did not permit controlling other variables that may have an impact on the studied relationships (e.g., cognitive status). Additionally, psychometric work on the NLEC is currently in progress. Both the SRMR fit index in the study by Sousa (2024) and the KR‐20 reliability estimates in the present study were suboptimal. Despite this, we retained negative life events as a covariate in the analysis, given the established link between this construct and depressive symptoms and attachment insecurity. Nonetheless, findings relying on this specific measure must be interpreted with prudence and would ideally benefit from future replication. Finally, data was assessed using self‐report measures only, which are susceptible to social desirability and provide a limited depth in our knowledge about the psychological constructs.

Future research that replicates current findings using longitudinal designs with longer duration and data assessment protocols that go beyond self‐report measures will be useful to further our knowledge about the present topics.

Additionally, it would be interesting to replicate the current findings using clinical and/or institutionalized samples. Formal care contexts (e.g., care homes, hospitals) often present circumstances that may interact with attachment insecurity and fear of self‐compassion to further impact depressive symptoms and quality of life. For example, older adults high in attachment anxiety may struggle to see themselves as worthy of the attention and support they receive from their formal/informal caregivers, and these individuals' increased sensitivity to abandonment may lead to hyperactivation behaviours that, in turn, may increase stress and compassion‐fatigue sentiments in those caregivers. In contrast, older adults high in attachment avoidance may downplay or neglect their own limitations and may struggle to engage with the formal/informal caregivers when truly needed. As such, providing psychological care that may help older adults to address their compassion‐related fears, as well as providing care to the caregivers themselves, seems like avenues for future research that are both needed and justifiable.

On a related note, future research that examines the other two flows of compassion (compassion towards others and compassion received from others) in relation to psychological adjustment will also be valuable. Returning to our previous example, these flows may play an especially important role in caregiving environments where older adults rely on social and professional support. As such, extending the present study's findings to clinical and care settings will be an important next step, offering valuable insights not only for older adults themselves but also for clinicians, care professionals and family caregivers.

Finally, it will be important to replicate the current findings across different countries and cultures. Portugal is generally characterized as a collectivist society that places a strong value on family cohesion and interdependence, with caregiving for older family members often being viewed as both a moral duty and a social expectation (Hofstede, 2001; Rego & Cunha, 2009). Such cultural norms may influence how attachment insecurity and (self)compassion‐related processes manifest in older adulthood. Therefore, future studies should explore whether the current findings hold in societies with different family structures, caregiving traditions and levels of individualism or collectivism.

## CONCLUSION

Notwithstanding these limitations, the present study is innovative, complements and expands previous literature and, as far as we know, is the first to investigate fear of self‐compassion specifically in older adults. Beyond its theoretical contributions, it also offers methodological advances. By employing a repeated‐measures design with three assessment points, it provides a stronger test of temporal relationships and mediating processes among the studied variables than could be achieved by previous cross‐sectional research.

Taken together, our findings highlight two main points. First, attachment (in)security remains of great importance in late life and may impact the psychological adjustment of older adults, directly and indirectly. Second, fear of self‐compassion should be considered when conducting research and/or intervention in this age‐group. The fear of being self‐compassionate may not only lead the individual to struggle to relate to themself in a kind and understanding manner, but also may prevent the positive outcomes associated with self‐compassion. Self‐compassion has shown a promising role in fostering positive ageing (Brown et al., 2019; Tavares et al., 2020), and is a malleable construct susceptible to being taught/learned and trained. Indeed, there is evidence that self‐compassion‐based interventions can benefit older adults (e.g., Perez‐Blasco et al., 2016). In particular, Poz and Craig (2025) conducted a recent review of the literature regarding Compassion‐Focused Therapy (CFT) specifically in this age‐group. A total of seven studies were reviewed and CFT was shown to be a viable and well‐received intervention for older adults, including those with general mental health issues and those with dementia. These findings suggest that both group and individual CFT might enhance mental health symptoms and overall well‐being in this population. Based on the present study's findings, we also emphasize that fear of self‐compassion, if left unchecked, may sabotage the efficacy of such interventions and possibly undermine them from the start.

At a time when most modern societies have a progressively ageing population, it is important to understand what influences the psychological (mal)adjustment of older adults and through which mechanisms, and what can be done to ensure a positive, healthy and dignified experience of late adulthood. Our study contributed to this objective and, hopefully, paved the way for future related research.

## AUTHOR CONTRIBUTIONS

**Lúcia Tavares:** Conceptualization; data curation; formal analysis; funding acquisition; methodology; project administration; writing – original draft. **Paula Vagos:** Conceptualization; methodology; supervision; validation; writing – review and editing. **Ana Xavier:** Conceptualization; methodology; validation; writing – review and editing; supervision.

## FUNDING INFORMATION

This research was supported by a grant received by Lúcia Tavares (Grant Number: 2022.13539.BD), sponsored by the Portuguese Foundation for Science and Technology (FCT), the European Social Fund (ESF) and the Por_Norte Program. The aforementioned funding sources had no involvement in the conduction of the research and/or preparation of the present article.

## CONFLICT OF INTEREST STATEMENT

The authors declare no potential conflicts of interest with respect to the research, authorship and/or publication of this article.

## ETHICS STATEMENT

Ethics approval for this study was obtained from the Ethics Committee for Health of Universidade Portucalense Infante D. Henrique, with the reference CES‐UPT‐01/05/21.

## PRIOR DISSEMINATION STATEMENT

The authors declare that the ideas and data appearing in the manuscript have not been disseminated before (e.g., at a conference or meeting, posted on a listserv, shared on a website). Additionally, this manuscript has not been published and is not under consideration for publication elsewhere.

## USE OF ARTIFICIAL INTELLIGENCE STATEMENT

The authors declare that no artificial intelligence tools were used in the conduction of this study, neither in the writing of the manuscript.

## Supporting information


Data S1.

## Data Availability

The data that support the findings of this study are available from Lúcia Tavares, upon reasonable request.
