# Supplementary material for: Attachment insecurity and psychological (mal)adjustment in older adults: The longitudinal role of fear of self‐compassion
Source: Psychol Psychother. 2026 Mar 29;99(3):931–45. doi: 10.1111/papt.70059 (PMC13456459; doi:10.1111/papt.70059)
Supplement: Supplementary file 1 [file PAPT-99-931-s001.docx]

**Supplementary Material**

**Mplus Syntax Files for Path Analyses**

**Model A – Initial**

TITLE: mediation_byFSC;

DATA: FILE IS mediation_byFSC.dat;

VARIABLE: NAMES ARE Anxiety Avoid FSC Depres QoL_tot;

ANALYSIS:

type = general;

bootstrap = 10000;

MODEL:

Depres ON Anxiety Avoid FSC;

QoL_tot ON Anxiety Avoid FSC;

FSC ON Anxiety Avoid;

MODEL INDIRECT:

Depres ind Anxiety;

Depres ind Avoid;

QoL_tot ind Anxiety;

QoL_tot ind Avoid;

OUTPUT: STDYX CINTERVAL (BCBOOTSTRAP);

**Model A – Respecified/Final**

TITLE: mediation_byFSC_final;

DATA: FILE IS mediation_byFSC.dat;

VARIABLE: NAMES ARE Anxiety Avoid FSC Depres QoL_tot;

ANALYSIS:

type = general;

bootstrap = 10000;

MODEL:

Depres ON FSC;

QoL_tot ON Anxiety Avoid FSC;

FSC ON Anxiety Avoid;

MODEL INDIRECT:

Depres ind Anxiety;

Depres ind Avoid;

QoL_tot ind Anxiety;

OUTPUT: STDYX CINTERVAL (BCBOOTSTRAP);

**Model B**

TITLE: mediation_byFSC_NLE as covariate;

DATA: FILE IS mediation_byFSC (NLE).dat;

VARIABLE: NAMES ARE NLE1 NLE2 NLE3 Anxiety Avoid FSC Depres QoL_tot;

ANALYSIS:

type = general;

bootstrap = 10000;

MODEL:

Depres ON NLE1 NLE2 NLE3 FSC;

QoL_tot ON NLE1 NLE2 NLE3 Anxiety Avoid FSC;

FSC ON NLE1 NLE2 Anxiety Avoid;

MODEL INDIRECT:

Depres ind Anxiety;

Depres ind Avoid;

QoL_tot ind Anxiety;

OUTPUT: STDYX CINTERVAL (BCBOOTSTRAP);
